# Temporal Changes in In Vivo Glutamate Signal during Demyelination and Remyelination in the Corpus Callosum: A Glutamate-Weighted Chemical Exchange Saturation Transfer Imaging Study

**DOI:** 10.3390/ijms21249468

**Published:** 2020-12-12

**Authors:** Do-Wan Lee, Hwon Heo, Chul-Woong Woo, Dong-Cheol Woo, Jeong-Kon Kim, Kyung-Won Kim, Dong-Hoon Lee

**Affiliations:** 1 Department of Radiology, Asan Medical Center, University of Ulsan College of Medicine, Seoul 05505, Korea; dwlee.mri@gmail.com (D.-W.L.); kim.jeongkon@gmail.com (J.-K.K.); medimash@gmail.com (K.-W.K.); 2 Department of Convergence Medicine, Asan Medical Center, University of Ulsan College of Medicine, Seoul 05505, Korea; heohwon@gmail.com (H.H.); dcwoo@amc.seoul.kr (D.-C.W.); 3 Convergence Medicine Research Center, Asan Institute for Life Sciences, Asan Medical Center, Seoul 05505, Korea; wandj79@hanmail.net; 4 Department of Radiation Convergence Engineering, College of Health Sciences, Yonsei University, Wonju, Gangwondo 26493, Korea

**Keywords:** glutamate, chemical exchange saturation transfer, demyelination, remyelination, cuprizone

## Abstract

Background: Glutamate-weighted chemical exchange saturation transfer (GluCEST) is a useful imaging tool that can be used to detect changes in glutamate levels in vivo and could also be helpful in the diagnosis of brain myelin changes. We investigated glutamate level changes in the cerebral white matter of a rat model of cuprizone-administered demyelination and remyelination using GluCEST. Method: We used a 7 T pre-clinical magnetic resonance imaging (MRI) system. The rats were divided into the normal control (CTRL), cuprizone-administered demyelination (CPZ_DM_), and remyelination (CPZ_RM_) groups. GluCEST data were analyzed using the conventional magnetization transfer ratio asymmetry in the corpus callosum. Immunohistochemistry and transmission electron microscopy analyses were also performed to investigate the myelinated axon changes in each group. Results: The quantified GluCEST signals differed significantly between the CPZ_DM_ and CTRL groups (−7.25 ± 1.42% vs. −2.84 ± 1.30%; *p* = 0.001). The increased GluCEST signals in the CPZ_DM_ group decreased after remyelination (−6.52 ± 1.95% in CPZ_RM_) to levels that did not differ significantly from those in the CTRL group (*p* = 0.734). Conclusion: The apparent temporal signal changes in GluCEST imaging during demyelination and remyelination demonstrated the potential usefulness of GluCEST imaging as a tool to monitor the myelination process.

## 1. Introduction

Myelin, which is present in the central nervous system, comprises an insulating sheath around the axons and promotes the efficient transmission of nerve impulses along the axon [1]. Demyelination is a condition in which myelin is lost along with the relative preservation of axons. It is caused by diseases that induce the death of oligodendrocytes, the cells that make and maintain the myelin sheath [2]. Multiple sclerosis (MS), a representative inflammatory demyelinating disease of the central nervous system, interferes with saltatory nerve conduction, leading to axonal degeneration and neurological dysfunction [3,4]. Remyelination is the opposite concept describing the restoration of the myelin sheath of demyelinated axons, saltatory conduction, and functional deficits [2]. As such, the evaluation and study of demyelination and remyelination may be a major factor in determining the outcome of myelin degeneration with substantial axonal and neuronal cell loss in diseases such as MS. It has become increasingly apparent in recent years that in vivo pathophysiological changes in glutamate occur during demyelination and remyelination in the brain’s white matter [5,6,7]. Therefore, the observation and evaluation of changes in glutamate levels, a potential essential biomarker, are important for estimating brain metabolism during myelination.

Chemical exchange saturation transfer (CEST) magnetic resonance imaging (MRI) has been introduced as a new contrast enhancement technique that enables the indirect detection of molecules with exchangeable endogenous protons and exchange-related properties [8]. In particular, among the endogenous agents, the phenomenon of solute-to-water proton exchange based on glutamate protons resonating at a well-known specific offset frequency from that of water has been widely applied in brain diseases, including tumors, ischemia, and psychiatric disorders [9,10,11,12,13]. Glutamate-weighted CEST (GluCEST) is usually applied using a high-field strength MRI system (≥7 Tesla) due to the fast exchange rate of glutamate [13,14]. Moreover, since a high B_0_ field increases the signal-to-noise ratio, which can be utilized for higher resolution, applying GluCEST imaging in clinical and pre-clinical systems above 7 T provides appropriate signal sensitivity.

This study investigated the in vivo changes in glutamate levels in the cerebral white matter using GluCEST imaging on a 7 T MRI system, on the premise that changes in glutamate may serve as a significant bio-imaging marker in the processes of demyelination and remyelination. Demyelination was modeled by applying a dietary cuprizone method (demyelination induced by the oral administration of the cuprizone copper chelator) to rats to induce demyelination in certain white matter tracts by toxins injurious to oligodendrocytes [15]. Thus, the simple cuprizone administration approach generally leads to demyelination of the white matter without any other specific modeling methods. Remyelination occurred through the interruption of the supply of cuprizone to remove toxins from the diet and by the simultaneous provision of normal feed.

## 2. Results

**Figure 1:**
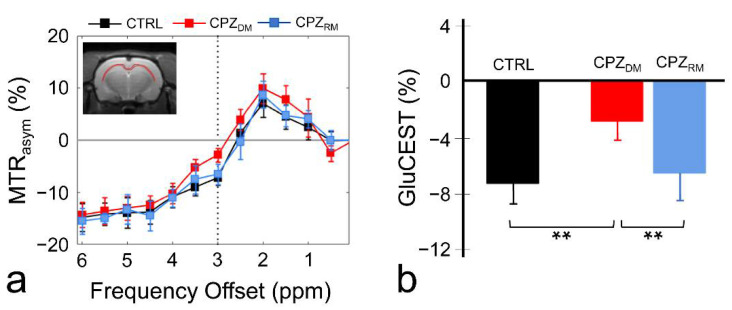
Magnetization transfer ratio asymmetry (MTR_asym_) spectra (**a**) in the corpus callosum region in the normal control (CTRL), demyelination (CPZ_DM_), and remyelination (CPZ_RM_) groups. The dotted line indicates the point at 3.0 ppm used for the quantification of glutamate-weighted chemical exchange saturation transfer (GluCEST). The quantified GluCEST signals (%) presented as bar graphs represent the mean + standard error of the mean for each group (*n* = 5–6 rats per group) (**b**). ** *p* < 0.01.

Figure 1 shows the magnetization transfer ratio asymmetry (MTR_asym_) spectra (a) and quantified GluCEST signals at 3.0 ppm (b) in each group. The MTR_asym_ spectra showed distinct differences between the CTRL and CPZ_DM_ groups, and after remyelination, no specific differences were observed between the CTRL and CPZ_RM_ groups. Overall, this indicated that the MTR_asym_ spectrum of the CPZ_DM_ group had a higher value than that of the CTRL and CPZ_RM_ groups as a function of frequency offset. Moreover, as the frequency offset increased (approximately ≥2.5 ppm), the MTR_asym_ spectra of all groups were negative, which was considered to be the effect of upfield nuclear Overhauser enhancement (NOE) (−2 to −5 ppm) [16,17]. The quantified GluCEST signals differed significantly between the CPZ_DM_ and CTRL groups (−7.25 ± 1.42% vs. −2.84 ± 1.30%), respectively (*p* = 0.001) (Figure 1b). However, after remyelination, the increased glutamate signals decreased in the CPZ_RM_ group (−6.52 ± 1.95%; *p* = 0.007) to levels that were not significantly different from those in the CTRL group (*p* = 0.734).

**Figure 2:**
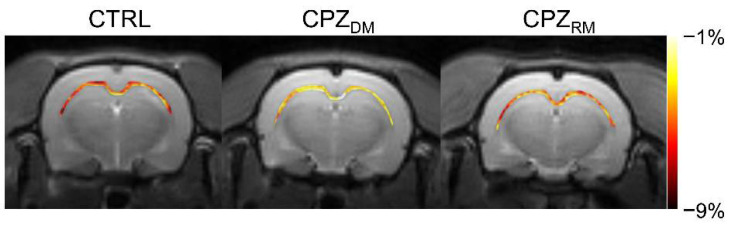
Reconstructed glutamate-weighted chemical exchange saturation transfer (GluCEST) maps overlaid on the unsaturated image targeting the corpus callosum region from representative rats from the normal control (CTRL), demyelination (CPZ_DM_), and remyelination (CPZ_RM_) groups.

Figure 2 shows the mapping results of GluCEST signals in representative rats, with a focus on the CC region and overlaid on the unsaturated CEST image in each group. The GluCEST signals in the CC region changed hyperintensities as demyelination progressed, compared to the normal control. Moreover, the GluCEST image contrast decreased again in the CPZ_RM_ group compared to that in the CPZ_DM_ group, which did not differ significantly from that in the CTRL group, as shown in Figure 1b.

**Figure 3:**
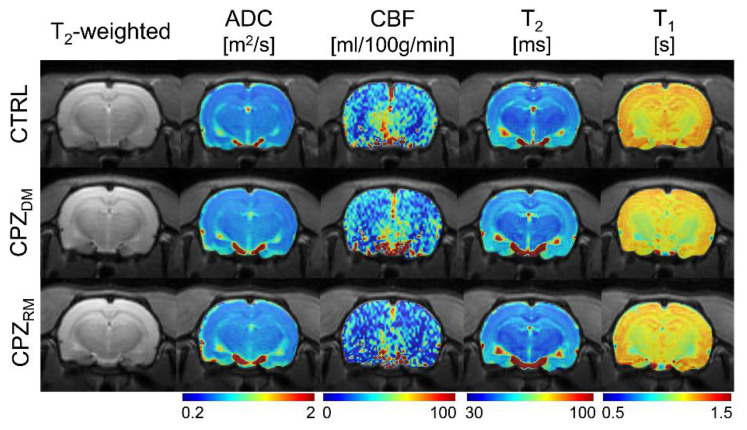
Reconstructed multi-parametric magnetic resonance (MR) images such as T_2_-weighted image, apparent diffusion coefficient (ADC), cerebral blood flow (CBF), and T_1_ and T_2_ maps in representative rats from the normal control (CTRL), demyelination (CPZ_DM_), and remyelination (CPZ_RM_) groups.

Figure 3 shows the reconstructed multi-parametric MR images of a representative rat in each group. The averaged multi-parametric values (ADC, CBF, T_2_, and T_1_) are shown in Table 1. As seen in the T_2_-weighted images, there were no specific signal differences in the CC area, suggesting a normal-appearing brain region even after demyelination. There were also no statistical differences among the groups (all *p* ≥ 0.505 in ADC, *p* ≥ 0.370 in CBF, *p* ≥ 0.390 in T_2_, and *p* ≥ 0.241 in T_1_).

**Figure 4:**
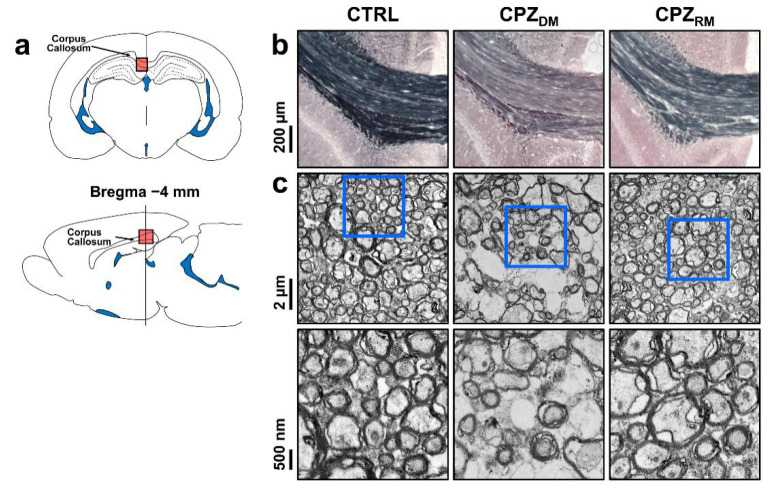
Cuprizone-induced demyelination and remyelination in the corpus callosum (CC). (**a**) Schematic representation of a coronal (**top**) and sagittal (**bottom**) section through a rat brain highlighting the middle segmentation of the CC that was examined. (**b**) Representative images of Black-Gold II-stained coronal sections of the middle CC in each group. Scale bar = 200 µm. (**c**) Representative electron micrographs (**top row**) and magnified images (**bottom row**) of the region of the CC indicating the ultrastructure of axonal myelination. Scale bar = 2 µm and 500 nm.

Figure 4 shows the histological validation in the CTRL, CPZ_DM_, and CPZ_RM_ groups. Representative Black-Gold II staining and TEM images show changes in the myelinated axons in the CC during the demyelination and remyelination phases. Seven weeks after cuprizone intoxication, the CC in the CPZ_DM_ group showed a markedly lower myelin density compared to those in the CTRL and CPZ_RM_ groups (Figure 4b). In addition, the CPZ_DM_ group showed demyelinated axons that were surrounded by thinner myelin sheaths than in CTRL and CPZ_RM_ (Figure 4c). After cuprizone withdrawal, we found that the substantially lower myelin content in the CPZ_DM_ group was reversed after 5 weeks of normal diet exposure. The CPZ_RM_ group showed axons wrapped with a new myelin sheath, resulting in similar axon and myelin morphologies compared to those in the CTRL group.

## 3. Discussion

In this study, we applied the GluCEST imaging technique, which provides high sensitivity to assess changes in glutamate levels in vivo, in a cuprizone-administered rat model to detect glutamate signal changes during demyelination and remyelination in the CC. Cuprizone may induce microscopic demyelination in normal-appearing brain tissue without the formation of macroscopic focal lesions, leading to stimulation of the ionotropic glutamate receptor, an excitatory toxic process in demyelination. Ultimately, this process may increase glutamate levels and lead to oligodendrocytic cell death [18]. Our results clearly showed in vivo glutamate changes in the demyelinated and remyelinated lesions, demonstrating increased GluCEST contrast, calculated as ((|GluCEST_CTRL_ − GluCEST_DM_|)/GluCEST_CTRL_ × 100) in the CPZ_DM_ group compared to those in the CTRL group (~60.8% increase). Notably, after the remyelination process, the GluCEST signal decreased again (~129.6% contrast decrease) to a level similar to that of the normal control (~10.1% difference in contrast between CTRL and CPZ_DM_).

As previously reported, glutamate concentrations are often increased in the early stages of demyelination in the brain white matter regions [6,19]. We also observed this phenomenon and hypothesized that the increased levels of glutamate in vivo are linked to glutamate excitotoxicity induced by inflammation [20]. Active inflammatory infiltrates were observed, in which a large amount of glutamate was produced and released by activated macrophages, microglial cells, and leukocytes [21]. In addition, it is worth noting that the glutamate signal exhibited by GluCEST imaging analysis, although the CC was a demyelinated region by cuprizone administration, showed no significant signal changes in multiparametric MR images in the present study. However, pathological changes in myelin sheaths during demyelination and remyelination were evident in the results of electron microscopy and myelin staining in this study. This phenomenon, which demonstrates normal-appearing white matter despite cuprizone-induced demyelination, is explained by the difficulty in observing the apparent lesion because it has not developed to the point where inflammatory molecules and tissue edema exist in the early stages of demyelination. Moreover, this phenomenon is often observed in human patients with MS. The pathological process by which glutamate levels increase in normal-appearing white matter regions occurs through the loss of receptor expression by oligodendrocytes in the lesion vicinity, which reduces glutamate uptake by more than 75% and results in ineffective glutamate removal [22]. Remyelination is also expressed as a result of an improvement in neuroaxonal integrity through the accumulation of oligodendrocyte progenitor cells (OPCs) in the CC and attenuates microglial activity and the beneficial role of reactive astrocytes [2]. We observed no significant differences in glutamate levels between the CTRL and CPZ_RM_ groups, similar to the results of a previous study.

Regarding the GluCEST signals quantified in the present study, we attempted to minimize the factors that affect GluCEST signal formation. Since B_0_ and B_1_ field inhomogeneities may affect signal drift by overall z-spectrum shift and inaccurate signal quantification, we applied WASSR and relative B_1_ map calculation to correct for field inhomogeneity in the GluCEST data, respectively. We also found no significant effect on the GluCEST signal through our evaluation of factors that can affect CEST signal formation, especially T_1_ and T_2_ values, while observing signal changes between the three groups (CTRL, CPZ_DM_, and CPZ_RM_) by multi-parametric MRI. In addition, we used a relatively high B_1_ saturation power and a short saturation time to observe glutamate-containing amine protons with medium/fast exchange rates in the 7 T system. Nevertheless, the negative GluCEST signal obtained from our results was likely due to the semi-solid MT effect, NOE, and immobile lipid signals combined in the MTR_asym_ spectrum [16,17]. This phenomenon was also revealed in the results of GluCEST imaging in human patients with MS at 7 T [14]. To obtain glutamate signals with less contamination by other effects, a further GluCEST signal quantification approach based on the signal fitting method should be applied beyond the current MTR_asym_-based quantification method [23,24,25].

Although our method showed the feasibility of a novel molecular MRI technique that can support the diagnosis and prognosis of demyelination and remyelination-related diseases where clinical symptoms are not conclusive, this study has several limitations and future research directions. First, to extend the anatomical coverage of GluCEST imaging for detecting signals in the demyelinated and remyelinated regions, multi-slice or three-dimensional GluCEST imaging should be applied. Further studies dealing with multiple brain regions, including different white matter regions, may provide information on the role of in vivo glutamate in myelin-related diseases as well as support current results. Second, contrary to our findings, other studies have indicated that glutamate levels might be reduced in demyelinated lesions, which can be attributed to mitochondrial dysfunction due to cuprizone administration [26]. In addition, a previous study showed that chronic exposure to cuprizone increased glutamate-aspartate transporter (GLAST) expression in the CC, suggesting that long-lasting changes in GLAST expression may be effective in coping with glutamate toxicity [27]. As such, there are various results and interpretations of changes in glutamate levels in in vivo studies of demyelination caused by cuprizone administration. Future studies using the chronic expression of demyelination and treatments for the remyelination process with various imaging, quantification, and biological analyses are needed to gain insight into the properties of in vivo glutamate changes. Moreover, this study was performed with a limited sample size, although our results showed that the change in glutamate level was significant between demyelinated and remyelinated lesions. Conducting further studies, with a larger number of subjects that model demyelination and remyelination-related diseases, would, we believe, provide results with more reliability. Finally, we need to confirm that the change in in vivo glutamate can occur before demyelination occurs, considering that the cuprizone-administered model is a toxic animal model in which demyelination is a secondary pathological change. Although we observed the GluCEST signal and pathological results for demyelination after seven weeks of cuprizone administration, evaluating the correlation between changes in GluCEST signals and histopathological samples periodically over a short period would be a meaningful study extending the current results.

## 4. Materials and Methods

### 4.1. Animal Models

All animal care and experiments were conducted with the approval of the Animal Care and Use Committee of Asan Medical Center of the University of Ulsan Medical School (approval date: 10-12-2018; permit code: 2018-13-271). Starting at four weeks of age, 18 male Sprague–Dawley rats were obtained from Orient Bio, Inc. (Seongnam, Kyunggi-do, Korea) and divided into the cuprizone-administered (*n* = 12) and normal control (CTRL, *n* = 6) groups. All twelve treatment rats were fed a milled diet containing 0.2% cuprizone (bis[cyclohexanone]oxaldihydrazone; Sigma–Aldrich, St. Louis, MO, USA), *ad libitum*, for seven weeks to induce demyelination [15,20,28]. Among the twelve rats, two died during cuprizone administration, so the demyelination and remyelination groups consisted of five rats each. After seven weeks, the demyelination model was considered complete, and five rats were randomly selected and sacrificed for histopathology (demyelination group, CPZ_DM_, *n* = 5). The remaining five rats were transferred to a normal chow diet for the recovery phase for an additional five weeks (remyelination group, CPZ_RM_, *n* = 5). The CTRL rats were fed a normal chow diet only.

### 4.2. MRI Data Acquisition

All MRI data acquisitions were conducted on a 7 T horizontal-bore PharmaScan 70/16 scanner (Bruker BioSpin, Ettlingen, Germany) with a 400 mT/m self-shielding gradient system. During imaging, the rat was maintained under anesthesia using 2.0% isoflurane delivered in a mixture of 75% air and 25% oxygen. Respiration and temperature were monitored using a small animal respiratory-gating system (SA Instruments Inc., Stony Brook, NY, USA) and a warm-water circulating flat-bed maintained at 37.0 ± 0.5 °C, respectively.

Region-of-interest (ROI)-based localized high-order shimming covering the whole brain area was applied before image scanning to achieve a homogeneous magnetic field in the MRI system. Multi-slice T_2_-weighted images were acquired using a turbo-rapid acquisition with relaxation enhancement (RARE) sequence with repetition time (TR) = 4 s, echo time (TE) = 33 ms, matrix size = 96 × 96, field of view (FOV) = 30 × 30 mm^2^, slice thickness = 1.5 mm, and RARE factor = 8. A single slice in which the hippocampus was well observed was then selected as the reference slice for GluCEST imaging. Single-slice GluCEST data were obtained using a fat-suppressed, turbo-RARE pulse sequence (TR = 4.2 s, TE = 36.4 ms, matrix size = 96 × 96, FOV = 30 × 30 mm^2^, slice thickness = 1.5 mm, RARE factor = 16) with 25 frequency offsets (−6 to +6 ppm at intervals of 0.5 ppm and unsaturated (S_0_) image). Images were acquired using a continuous-wave radiofrequency (RF) saturation pulse (power = 3.6 μT, and time = 1 s) [12,13]. A water saturation shift referencing (WASSR) dataset [29] with 29 frequency offsets was acquired from −0.8 to 0.8 ppm (0.05 ppm increments) using 0.3 μT RF saturation power for B_0_ correction. A B_1_ map using a double flip-angle (30° and 60°) was also acquired for B_1_ correction [13].

Additionally, based on the same GluCEST image slice, we also acquired multi-parametric MRI data as follows: (i) apparent diffusion coefficient (ADC) maps were acquired using a single-shot spin-echo echo planar imaging sequence with seven b-values (0, 166.7, 333.3, 500, 666.7, 833.3, and 1000 s/mm^2^), TR = 3 s, TE = 18.7 ms, and NA = 3; (ii) cerebral blood flow (CBF) maps were obtained using a flow-sensitive alternating inversion recovery (arterial spin labeling with variable inversion times (TIs)) (16 TIs: TI_1_ = 35 ms, TI_2_ to TI_15_ = 100 to 1400 ms (100 ms increment), and TI_16_ = 1600 ms, TR = 10,212.2–11,777.2 ms, TE = 36.36 ms, and NA = 1); (iii) T_1_ relaxation maps were obtained using RARE with a variable repetition time sequence with six TRs (0.6, 0.9, 1.5, 2.5, 4.0, and 7.0 s), TE = 12.2 ms, RARE factor = 4, NA = 1; and (iv) T_2_ relaxation maps were obtained using a multi-spin multi-echo sequence with 15 TEs (10 to 150 ms with 10 ms increments), TR = 3 s, and NA = 1.

### 4.3. MRI Data Analysis

Before GluCEST imaging analysis, B_0_ and B_1_ corrections were applied using the WASSR method and relative B_1_ values were calculated on the B_1_ map. Using B_0_- and B_1_-corrected CEST data, the GluCEST contrast was computed by subtracting the normalized magnetization signal at 3.0 ppm from the magnetization at the corresponding reference frequency symmetrically upfield from water; GluCEST (%) = 100 × [S_sat_(−3.0 ppm) − S_sat_(+3.0 ppm)]/S_sat_(−3.0 ppm) [13]. An ROI for the calculation of the GluCEST values was manually drawn in the corpus callosum (CC) region. We also applied the same ROI in each rat for multi-parametric MR data analysis. The T_1_ and T_2_ relaxation maps were reconstructed using the following equations: I(t) = I_0_·[1 − C·exp(−TR/T_1_)] and I(t) = I_0_·exp(−TE/T_2_), respectively. The ADC map was fitted using the following equation: I = I_0_·exp(−b·ADC). The CBF map was reconstructed from images with and without labeling. Statistical differences in the calculated MR signals, including GluCEST and multiparametric MRIs among the three groups (CTRL, CPZ_DM_, and CPZ_RM_), were analyzed by one-way analysis of variance (ANOVA), followed by Tukey’s post hoc tests. The level of significance was set at *p* < 0.05. All MRI data analyses were performed using MATLAB R2019b (The MathWorks, Natick, MA, USA) and statistical analysis was performed using PASW Statistics for Windows, version 18.0 (SPSS Inc., Chicago, IL, USA).

### 4.4. Transmission Electron Microscopy (TEM)

In a subset of rats, demyelination and remyelination of the CC were observed at the ultrastructural level by transmission electron microscopy (TEM). Rats were anesthetized and fixed by cardiac perfusion using 4.0% paraformaldehyde (PFA). The harvested brain samples encompassing the midline of the CC were placed in a cold fixative solution with 2.5% glutaraldehyde in phosphate-buffered saline (PBS, 0.1 M, pH = 7.4) at 4 °C for 4 h. The brains were then dissected into small pieces following the midline sections and oriented such that cross-sections of axons within the CC were obtained. After rinsing with PBS, the specimens were postfixed in 1% osmium tetroxide solution for 1 h. The samples were then rinsed again using PBS. The tissues were dehydrated in a series of ascending ethanol solutions and infiltrated with propylene oxide. Then, pure Epon resin was used for embedding and the samples were incubated at 60 °C for 72 h. Semi-thin sections (thickness = 1 μm) were stained with toluidine blue to identify the target orientation and location of the sections under a microscope. Ultrathin serial sections were prepared from the center of the embedded blocks containing the axons in the CC area using an ultramicrotome (thickness = 60 nm). The sections were then placed on copper slot grids and stained with 2% uranyl acetate and lead citrate. All sections were examined under a Hitachi H-7600 TEM at 80 kV.

### 4.5. Myelin Staining

To visualize the myelin distribution in the CC, the whole brain was sectioned from the bregma −4 mm in the coronal plane at 20 μm thickness and then rehydrated in distilled Milli-Q water for 2 min. The slide sections were incubated in a 0.3% solution of Black-Gold II (AG105, Millipore Corp, Billerica, MA, USA), dissolved in 0.9% saline, and heated to 60 °C for 12 min. All slides were then washed twice with Milli-Q water. All transferred slides were fixed in a 1% sodium thiosulfate solution for 3 min and then dehydrated using a series of graduated ethanol solutions, cleared in xylene for 2 min, and cover-slipped with mounting media. All sections were examined under a Zeiss Axio Observer.Z1 bright field microscope.

## 5. Conclusions

The present study represents the feasibility and potential of GluCEST imaging to monitor in vivo glutamate levels in the demyelination and remyelination processes. Notably, the GluCEST image contrast between the demyelinated and remyelinated lesions was increased by the change in the concentration of glutamate in vivo. Our results suggest that the GluCEST signal may be a valuable MRI biomarker to identify demyelinated and remyelinated lesions and may be a potential tool for verifying the effectiveness of diagnosis and treatment in cases of demyelinated and remyelinated diseases.

## Figures and Tables

**Table 1:** Calculated multi-parametric magnetic resonance (MR) signal intensities. All values are represented as means ± standard deviation.

	ADC (m^2^/s)	CBF (ml/100g/min)	T_2_ (ms)	T_1_ (s)
Normal control (CTRL)	0.63 ± 0.01	25.04 ± 8.21	45.48 ± 1.25	1.14 ± 0.02
Demyelination (CPZ_DM_)	0.64 ± 0.03	29.96 ± 7.23	46.63 ± 1.18	1.13 ± 0.03
Remyelination (CPZ_RM_)	0.64 ± 0.01	22.96 ± 8.13	46.02 ± 1.73	1.12 ± 0.02

ADC: apparent diffusion coefficient; CBF: cerebral blood flow.

## References

[1] Oakden W., Bock N.A., Al-Ebraheem A., Farquharson M.J., Stanisz G.J. (2017). Early regional cuprizone-induced demyelination in a rat model revealed with MRI. NMR Biomed..

[2] Franklin R.J., Ffrench-Constant C. (2008). Remyelination in the CNS: From biology to therapy. Nat. Rev. Neurosci..

[3] Azevedo C.J., Kornak J., Chu P., Sampat M., Okuda D.T., Cree B.A., Nelson S.J., Hauser S.L., Pelletier D. (2014). In vivo evidence of glutamate toxicity in multiple sclerosis. Ann. Neurol..

[4] Geurts J.J., Reuling I.E., Vrenken H., Uitdehaag B.M., Polman C.H., Castelijns J.A., Barkhof F., Pouwels P.J. (2006). MR spectroscopic evidence for thalamic and hippocampal, but not cortical, damage in multiple sclerosis. Magn. Reson. Med..

[5] Tisell A., Leinhard O.D., Warntjes J.B., Aalto A., Smedby O., Landtblom A.M., Lundberg P. (2013). Increased concentrations of glutamate and glutamine in normal-appearing white matter of patients with multiple sclerosis and normal MR imaging brain scans. PLoS ONE.

[6] Srinivasan R., Sailasuta N., Hurd R., Nelson S., Pelletier D. (2005). Evidence of elevated glutamate in multiple sclerosis using magnetic resonance spectroscopy at 3 T. Brain J. Neurol..

[7] Pitt D., Werner P., Raine C.S. (2000). Glutamate excitotoxicity in a model of multiple sclerosis. Nat. Med..

[8] van Zijl P.C.M., Yadav N.N. (2011). Chemical Exchange Saturation Transfer (CEST): What is in a Name and What Isn’t?. Magn. Reson. Med..

[9] Roalf D.R., Nanga R.P.R., Rupert P.E., Hariharan H., Quarmley M., Calkins M.E., Dress E., Prabhakaran K., Elliott M.A., Moberg P.J. (2017). Glutamate imaging (GluCEST) reveals lower brain GluCEST contrast in patients on the psychosis spectrum. Mol. Psychiatry.

[10] Davis K.A., Nanga R.P., Das S., Chen S.H., Hadar P.N., Pollard J.R., Lucas T.H., Shinohara R.T., Litt B., Hariharan H. (2015). Glutamate imaging (GluCEST) lateralizes epileptic foci in nonlesional temporal lobe epilepsy. Sci. Transl. Med..

[11] Zhou R., Bagga P., Nath K., Hariharan H., Mankoff D.A., Reddy R. (2018). Glutamate-Weighted Chemical Exchange Saturation Transfer Magnetic Resonance Imaging Detects Glutaminase Inhibition in a Mouse Model of Triple-Negative Breast Cancer. Cancer Res..

[12] Lee D.H., Woo C.W., Kwon J.I., Chae Y.J., Ham S.J., Suh J.Y., Kim S.T., Kim J.K., Kim K.W., Woo D.C. (2019). Cerebral mapping of glutamate using chemical exchange saturation transfer imaging in a rat model of stress-induced sleep disturbance at 7.0T. J. Magn. Reson. Imaging.

[13] Cai K.J., Haris M., Singh A., Kogan F., Greenberg J.H., Hariharan H., Detre J.A., Reddy R. (2012). Magnetic resonance imaging of glutamate. Nat. Med..

[14] O’Grady K.P., Dula A.N., Lyttle B.D., Thompson L.M., Conrad B.N., Box B.A., McKeithan L.J., Pawate S., Bagnato F., Landman B.A. (2019). Glutamate-sensitive imaging and evaluation of cognitive impairment in multiple sclerosis. Mult. Scler..

[15] Nathoo N., Yong V.W., Dunn J.F. (2014). Understanding disease processes in multiple sclerosis through magnetic resonance imaging studies in animal models. Neuroimage Clin..

[16] Liu D.P., Zhou J.Y., Xue R., Zuo Z.T., An J., Wang D.J.J. (2013). Quantitative Characterization of Nuclear Overhauser Enhancement and Amide Proton Transfer Effects in the Human Brain at 7 Tesla. Magn. Reson. Med..

[17] Heo H.Y., Jones C.K., Hua J., Yadav N., Agarwal S., Zhou J.Y., van Zijl P.C.M., Pillai J.J. (2016). Whole-Brain Amide Proton Transfer (APT) and Nuclear Overhauser Enhancement (NOE) Imaging in Glioma Patients Using Low-Power Steady-State Pulsed Chemical Exchange Saturation Transfer (CEST) Imaging at 7T. J. Magn. Reson. Imaging.

[18] Matute C., Alberdi E., Domercq M., Sanchez-Gomez M.V., Perez-Samartin A., Rodriguez-Antiguedad A., Perez-Cerda F. (2007). Excitotoxic damage to white matter. J. Anat..

[19] Khodanovich M.Y., Sorokina I.V., Glazacheva V.Y., Akulov A.E., Nemirovich-Danchenko N.M., Romashchenko A.V., Tolstikova T.G., Mustafina L.R., Yarnykh V.L. (2017). Histological validation of fast macromolecular proton fraction mapping as a quantitative myelin imaging method in the cuprizone demyelination model. Sci. Rep..

[20] Orije J., Kara F., Guglielmetti C., Praet J., Van der Linden A., Ponsaerts P., Verhoye M. (2015). Longitudinal monitoring of metabolic alterations in cuprizone mouse model of multiple sclerosis using 1H-magnetic resonance spectroscopy. Neuroimage.

[21] Piani D., Frei K., Do K.Q., Cuenod M., Fontana A. (1991). Murine Brain Macrophages Induce Nmda Receptor Mediated Neurotoxicity in vitro by Secreting Glutamate. Neurosci. Lett..

[22] Pitt D., Nagelmeier I.E., Wilson H.C., Raine C.S. (2003). Glutamate uptake by oligodendrocytes: Implications for excitotoxicity in multiple sclerosis. Neurology.

[23] Zaiss M., Xu J., Goerke S., Khan I.S., Singer R.J., Gore J.C., Gochberg D.F., Bachert P. (2014). Inverse Z-spectrum analysis for spillover-, MT-, and T1 -corrected steady-state pulsed CEST-MRI--application to pH-weighted MRI of acute stroke. NMR Biomed..

[24] Singh A., Debnath A., Cai K., Bagga P., Haris M., Hariharan H., Reddy R. (2019). Evaluating the feasibility of creatine-weighted CEST MRI in human brain at 7 T using a Z-spectral fitting approach. NMR Biomed..

[25] Debnath A., Hariharan H., Nanga R.P.R., Reddy R., Singh A. (2020). Glutamate-Weighted CEST Contrast After Removal of Magnetization Transfer Effect in Human Brain and Rat Brain with Tumor. Mol. Imaging Biol..

[26] Praet J., Orije J., Kara F., Guglielmetti C., Santermans E., Daans J., Hens N., Verhoye M., Berneman Z., Ponsaerts P. (2015). Cuprizone-induced demyelination and demyelination-associated inflammation result in different proton magnetic resonance metabolite spectra. NMR Biomed..

[27] Azami Tameh A., Clarner T., Beyer C., Atlasi M.A., Hassanzadeh G., Naderian H. (2013). Regional regulation of glutamate signaling during cuprizone-induced demyelination in the brain. Ann. Anat..

[28] Wang N., Zhuang J., Wei H.J., Dibb R., Qi Y., Liu C.L. (2019). Probing demyelination and remyelination of the cuprizone mouse model using multimodality MRI. J. Magn. Reson. Imaging.

[29] Kim M., Gillen J., Landman B.A., Zhou J., van Zijl P.C. (2009). Water saturation shift referencing (WASSR) for chemical exchange saturation transfer (CEST) experiments. Magn. Reson. Med..

